# Survival and hepatitis status among Asian Americans with hepatocellular carcinoma treated without liver transplantation

**DOI:** 10.1186/1471-2407-9-46

**Published:** 2009-02-04

**Authors:** Jessica P Hwang, Manal M Hassan

**Affiliations:** 1 Department of General Internal Medicine, Ambulatory Treatment and Emergency Care, The University of Texas M. D. Anderson Cancer Center, Houston, Texas, USA; 2 Department of Gastrointestinal Medical Oncology, The University of Texas M. D. Anderson Cancer Center, Houston, Texas, USA

## Abstract

**Background:**

Chronic hepatitis B virus (HBV) and hepatitis C virus (HCV) are established causes of HCC. HCC patients are often diagnosed late and receive palliative therapies, however, the survival of Asian American patients with HCC treated without transplantation has not been well studied. We reviewed our institution's experience to determine predictors and rates of survival in Asian American HCC patients treated without transplantation.

**Methods:**

We identified Asian American patients with HCC referred to M. D. Anderson Cancer Center. Patients were tested for HBV and HCV. Survival curves were generated by Kaplan-Meier method. Multivariate Cox proportional hazards regression was used to test the relationship between prognostic factors and survival.

**Results:**

Of 82 Asian American HCC patients, most had advanced disease (65%) and received treatment (68%); however, only 11% had surgical resection. 94% had positive anti-HBc and 61% had positive HBsAg. 20% had positive anti-HCV. There were no significant changes in the rates of HBV and HCV over time. Male gender, high alpha-fetoprotein levels, and stage IV disease were associated with shorter survival Overall median survival was 9.2 months (95% CI 6.5–11.9), and the survival of HCV and HBV patients was not statistically different.

**Conclusion:**

The survival rate of Asian American patients with advanced HCC, for whom transplantation was not available, was low. Timely hepatitis screening and interventions by primary care physicians may be the most logical solution to reduce the burden of hepatitis-associated HCC among Asian Americans.

## Background

Hepatocellular carcinoma (HCC), one of the most common cancers worldwide, is rarely detected early and is usually fatal within months of diagnosis. Recent reports have shown significant increases in the incidence of HCC in the US during the past two decades [1,2].

Chronic hepatitis B virus (HBV) and hepatitis C virus (HCV) infections are established causes of HCC [3]. Worldwide, 53% of HCC cases are attributed to HBV and 25% are attributed to HCV [4]. Early detection and treatment of HBV and HCV can lead to improved surveillance for HCC, thus resulting in earlier detection of HCC and reduction in HCC-associated mortality. Screening for HBV and HCV is routine in Asian countries such as Japan and other developed eastern countries with high prevalence of these diseases [5], but screening among Asian Americans is currently is not widespread in the US [6].

Although Asian Americans represent only 4.8% of the US population [7], cross-sectional studies from screening events in large metropolitan areas with significant Asian American communities indicate that the prevalence of chronic HBV among Asian Americans is 8.9% to 15%, making Asian Americans the racial or ethnic group with the highest HBV prevalence in the US [8-10]. However, the true prevalence of chronic HBV among Asian Americans remains unclear because population-based studies have included only small numbers of Asian Americans.

Information on the prevalence of HCC among Asian Americans is sparse. A large retrospective study at an urban hospital in California estimated the prevalence of HCV among Asian Americans to be approximately 8% [11]. Globally, the incidence of HCC is increasing in many countries, and this has been attributed to an increase in HCV-related HCC [12,13].

When HCC is detected early, it can be treated with curative modalities, including liver transplantation and hepatic resection. Tumor resectability has been shown to be an independent predictor of improved survival among patients with HCC [14]. However, most patients with HCC are diagnosed at a late stage and are not candidates for radical, curative therapies [15]; they are treated instead with palliative treatments such as systemic chemotherapy, arterial chemoembolism, or hormonal therapy. Previous studies have examined clinical outcomes of Asian American patients with HCC, but these studies have included patients for whom transplantation was an option [16-18]. The survival of Asian American patients with HCC treated without transplantation has not previously been described. At our tertiary cancer center, we do not offer liver transplantation, and most patients with HCC receive palliative therapies.

A previous study of Asian Americans with HCC has shown that HBV infection predicts decreased survival [18] whereas another study showed that hepatitis infection did not affect survival [17]. To better understand the impact of chronic HBV and HCV on Asian American patients with advanced HCC, we reviewed our institution's experience to determine the predictors and rates of survival in Asian American HCC patients treated without transplantation.

## Methods

### Patient population

Using the tumor registry of The University of Texas M. D. Anderson Cancer Center, we obtained a list of all patients who reported themselves as being of Asian American ethnicity with a diagnosis of HCC who presented at M. D. Anderson Cancer Center between January 1992 and December 2005. All patients had a liver biopsy which showed pathologic confirmation of HCC. This retrospective research protocol was reviewed and approved by the M. D. Anderson institutional review board which granted a waiver of informed consent.

The identified patients' medical records were reviewed for demographic, clinical, and risk factor information. Structured data were collected from forms that are used to collect data from patients during their first visit to M. D. Anderson and are kept as part of the medical record. These forms have been validated by the Departments of Epidemiology and Gastrointestinal Medical Oncology at M. D. Anderson. In addition to demographic information, these forms include detailed questions on smoking, alcohol use, family history of cancer, and medical history. For this study, use of alcohol was defined as the consumption of at least 4 drinks monthly for 6 months.

At M. D. Anderson, all patients with a diagnosis of HCC are examined by a hepatobiliary oncologist for signs of cirrhosis, such as manifestations related to portal hypertension (e.g., ascites, bleeding from esophageal varices, and hepatic encephalopathy); palmar erythema; spider angioma; and finger clubbing. Records of these examinations, plus pathologic assessments and computed tomography scans, were reviewed for evidence of liver cirrhosis. The patients' medical records were also reviewed for information on hepatitis status. All patients had been tested for the presence of antibody to hepatitis C virus (anti-HCV), hepatitis B surface antigen (HBsAg), and antibody to hepatitis B core antigen (anti-HBc) with a second-generation enzyme-linked immunosorbent assay (Abbott Laboratories, North Chicago, IL), and samples yielding a positive test result were tested three times to confirm the results.

### Statistical methods

Descriptive statistics were used to compare the proportion of demographic and clinical factors between HBV-infected patients with and without HCV infection. SPSS (SPSS, Inc., Chicago, IL, 2003) was used for all data management and statistical analysis. Baseline laboratory markers were expressed as medians. Survival curves were generated by the Kaplan-Meier method, and the statistical significance of potential differences between groups was determined by using Gehan's modification of the Wilcoxon signed-rank test. Multivariate Cox proportional hazards regression was used to test the relationship between various prognostic factors and survival in all patients. Only those variables showing a statistically significant (p ≤ 0.05) relationship with survival in univariate analyses were included in the overall multivariate Cox model. The variables tested included age, sex, the presence or absence of cirrhosis, serologic evidence of HBV or HCV infection, comorbid conditions, and stage of HCC, previous treatment, and family history of cancer. Alpha-fetoprotein level, pathologic tumor type, and alcohol and tobacco use were also included. The assumptions of the Cox regression analyses were verified, and all reported p values were from two-sided tests.

## Results

### Patient characteristics

From January 1992 through December 2005, 82 Asian Americans with HCC were referred to M. D. Anderson. Overall, these patients represented 10% of all patients with HCC referred to M. D. Anderson during the study period. Most (81.1%) of the Asian American patients with HCC were residents of the US. Other demographic characteristics of the subjects are shown in Table 1. Mean age ± standard deviation was 57.8 ± 12.4 years, and the male-to-female ratio was greater than 2:1. Most of the patients (74.3%) were originally from Vietnam, China, or Korea. About 43% were not proficient in the English language during the clinical visits and needed language assistance. Thirty-eight patients (46%) were referred to M. D. Anderson between 1992 and 1998, and 44 (54%) were referred between 1999 and 2005.

**Table 1:** Demographic Characteristics of Asian American HCC patients by HBV and HCV status

Characteristics	All Patients N = 82 (%)	^1^HBV^+^/HCV^- ^ N = 63 (%)	^2^HCV^+ ^± HBV^+^ N = 16 (%)
**Age group**			
30–41	8 (9.8)	8 (12.7)	0
41–50	19 (23.2)	19 (30.2)	0
51–60	21 (25.6)	17 (27)	4 (25)
61–70	19 (23.2)	11(17.5)	6 (37.5)
>70	15 (18.3)	8 (12.7)	6 (37.5)
			
**Sex**			
Male	57 (69.5)	47 (74.6)	9 (56.3)
Female	25 (30.5)	16 (25.4)	7 (43.8)
			
**Country of origin**			
Cambodia	1 (1.2)	1 (1.6)	0
China	13 (15.9)	10 (15.9)	2 (12.5)
Hong Kong	4 (4.9)	3 (4.8)	0
Korea	13 (15.9)	11 (17.5)	2 (12.5)
Philippines	6 (7.3)	6 (9.5)	0
Taiwan	8 (9.8)	6 (9.5)	2 (12.5)
Thailand	2 (2.4)	2 (3.2)	0
Vietnam	35 (42.7)	24 (38.1)	10 (62.5)
			
**Use of English translator**			
Yes	35 (42.7)	21 (33.3)	12 (75)
No	47 (57.3)	42 (66.7)	4 (25)

^1^HBV^+^/HCV^- ^represents patients with HBsAg^+^/anti-HBc^+^/anti-HCV^- ^or HBsAg^-^/anti-HBc^+^/anti-HCV^-^

^2^HCV^+ ^± HBV^+ ^represents patients with anti-HCV^+^/HBsAg^-^/anti-HBc^-^, anti-HCV^+^/HBsAg^-^/anti-HBc^+^, or anti-HCV^+^/HBsAg^+^/anti-HBc^+^

Clinical characteristics are shown on Table 2. The majority of patients had advanced-stage HCC and significant liver damage. Most patients had stage IV disease at the time of their initial presentation to M. D. Anderson, and 21% had stage IVB disease (distant metastases). Most patients had alpha-fetoprotein levels of at least 100 ng/mL, and 46% had cirrhosis. Nearly two thirds (68.4%) of the patients ever received therapy for HCC either prior to or at M. D. Anderson, but only 11% (n = 9) had surgical resection at M. D. Anderson.

**Table 2:** Clinical and Epidemiological Characteristics of Asian American HCC patients

Characteristics	All Patients N = 82 (%)	^1^HBV^+^/HCV^-^ N = 63 (%)	^2^HCV^+ ^± HBV^+^ N = 16 (%)
**TNM stage**			
I or II	11 (13.4)	6 (9.7)	4 (25)
III	18 (22.0)	13 (21)	5 (31.3)
IV	53 (64.6)	43 (69.3)	7 (43.8)
			
**Ever treated**	55 (67.1)	43 (68.3)	11 (68.8)
			
**Treatment type at M. D. Anderson Cancer Center**			
None	32 (39.1)	25 (39.7)	5 (31.3)
Chemotherapy	39 (47.6)	29 (46)	9 (56.3)
Surgery	4 (4.9)	4 (6.3)	0
Chemotherapy & surgery	5 (6.1)	3 (4.8)	2 (12.5)
Other	2 (2.4)	2 (3.2)	0
			
**Cirrhosis**	38 (46.3)	28 (44.4)	10 (62.5)
			
**AFP level (ng/mL)**			
< 100	29 (35.4)	21 (35)	7 (46.7)
≥ 100	53 (64.6)	39 (65)	8 (53.3)
			
^3^**Tumor differentiation**			
Well/Moderately differentiated	67 (82.7)	50 (80.6)	14 (87.5)
Poorly differentiated	13 (16.0)	11 (17.7)	2 (12.5)
			
^4^**HCC risk factors**			
Diabetes mellitus	9 (11.0)	6 (9.8)	3 (18.8)
Cigarette smoking	36 (43.9)	28 (45.9)	8 (50)
Alcohol use	28 (34.1)	20 (32.8)	8 (50)
Family history of cancer	42 (51.2)	34 (55.7)	6 (37.5)
Family history of liver cancer	18 (22.0)	17 (27.9)	1 (6.3)
			
**HBV/HCV status**			
None	3 (3.7)	___	___
HBsAg^+^/anti-HBc^+^/anti-HCV^-^	48 (58.5)	___	___
HBsAg^-^/anti-HBc^+/^anti-HCV^-^	15 (18.3)	___	___
HBsAg^-^/anti-HBc^-^/anti-HCV^+^	2 (2.4)	___	___
HBsAg^-^/anti-HBc^+^/anti-HCV^+^	12 (14.6)	___	___
HBsAg^+^/anti-HBc^+^/anti-HCV^+^	2 (2.4)		

^1^HBV^+^/HCV^- ^represents patients with HBsAg^+^/anti-HBc^+^/anti-HCV^- ^or HBsAg^-^/anti-HBc^+^/anti-HCV^-^

^2^HCV^+ ^± HBV^+ ^represents patients with anti-HCV^+^/HBsAg^-^/anti-HBc^-^, anti-HCV^+^/HBsAg^-^/anti-HBc^+^, or anti-HCV^+^/HBsAg^+^/anti-HBc^+^

^3^The N for tumor differentiation does not equal total number because of missing data for one HBV^+^/HCV^- ^patient.

^4^The N for HCC risk factors does not equal total number because of missing data for 2 HBV^+^/HCV^- ^patients.

### Rates of chronic HBV and HCV infection

The majority of patients (n = 77, 93.9%) had evidence of previous exposure to HBV as demonstrated by a positive HBsAg (n = 50, 61%) or an isolated positive anti-HBc (n = 27, 32.9%) test result (Table 2). 63 patients had prior infection with HBV alone, as evidence by a positive HBsAg or anti-HBc test result, and did not have HCV. Sixteen HCC patients (19.5%) were infected with HCV as indicated by a positive anti-HCV test result, where 2 patients were infected with HCV alone, and 14 patients had dual infection with HBV and HCV. Due to small numbers, these 16 patients are classified together as HCV+ with or without HBV (Tables 1, 2). For clarity, these 16 patients will be referred to as HCV+ patients in the text. Three patients had no evidence of hepatitis infection.

**Figure 1:**
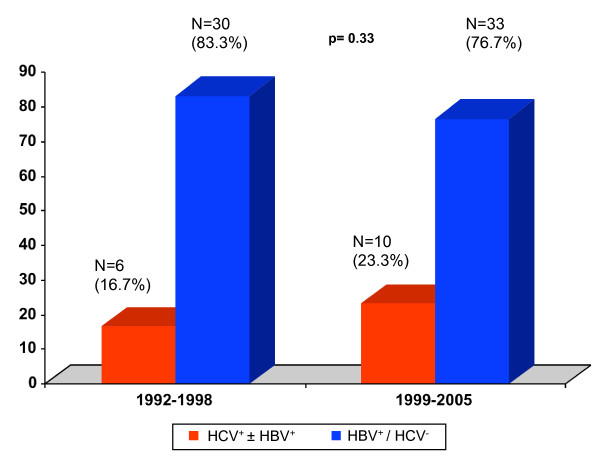
**Proportions of patients with HCC whose disease was associated with HCV or HBV by era of initial presentation**. HBV^+^/HCV^- ^represents patients with HBsAg^+^/anti-HBc^+^/anti-HCV^- ^or HBsAg^-^/anti-HBc^+^/anti-HCV^-^. HCV^+ ^± HBV^+ ^represents patients with anti-HCV^+^/HBsAg^-^/anti-HBc^-^, anti-HCV^+^/HBsAg^-^/anti-HBc^+^, or anti-HCV^+^/HBsAg^+^/anti-HBc^+^.

**Figure 2:**
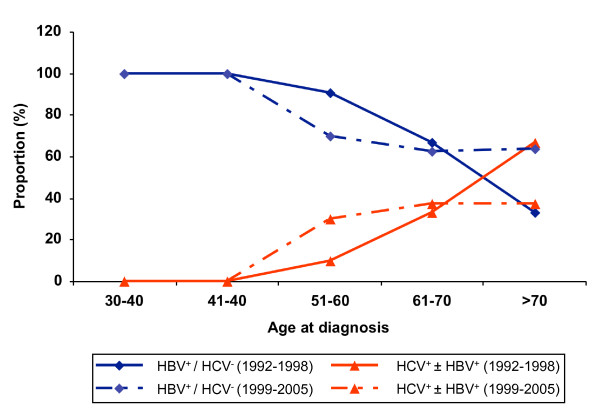
**Proportions of patients with HCC whose disease was associated with HCV or HBV by age group and era of presentation**. HBV^+^/HCV^- ^represents patients with HBsAg^+^/anti-HBc^+^/anti-HCV^- ^or HBsAg^-^/anti-HBc^+^/anti-HCV^-^. HCV^+ ^± HBV^+ ^represents patients with anti-HCV^+^/HBsAg^-^/anti-HBc^-^, anti-HCV^+^/HBsAg^-^/anti-HBc^+^, or anti-HCV^+^/HBsAg^+^/anti-HBc^+^.

The proportion of HCC patients infected with HBV infection alone was similar among patients presenting in 1992–1998 (n = 30, 83.3%) compared to those presenting in 1999–2005 (n = 33, 76.7%, p = 0.33) (Figure 1). Though numbers are small, the proportion of HCC patients with HCV infection may have increased slightly over time, from 16.7% in 1992–1998 to 23.3% in 1999–2005, but this difference was not significant (p = 0.58). A slightly higher proportion of HCC patients infected with HCV was observed among patients older than 50 years than among younger patients, particularly in the later period (1999–2005) (Figure 2). A substantial majority of Asian American patients with HCC were also infected with HBV, and this was true across all age groups and during both time intervals (Figures 1 and 2).

### Patient characteristics by hepatitis status

HCC patients with HBV infection were younger than HCC patients with HCV infection; 87.3% of the HBV patients were 41 to 60 years of age, whereas 75% of the HCV patients were 61 years of age or older (Table 1). HBV patients were also more likely to present with stage IV disease (69.3% vs. 43.8%) and had higher rates of not receiving any treatment (39.7% vs. 31.3%) than patients infected with HCV. As expected, more HCV patients had cirrhosis (62.5% vs. 44.4%). In both the HBV and HCV groups, high proportions of patients described their country of origin as Vietnam. Three-fourths of the HCV patients needed translation services, compared with 33% of the HBV patients (Table 1).

### Prevalence of other known risk factors for HCC

Table 2 shows the distribution of the other major risk factors for HCC, besides hepatitis, in this highly selected population. Overall, more than half of the patients in this study had a family history of cancer, which may have included a first- or second-degree relative with HCC. HCV patients had higher rates of diabetes mellitus, cigarette smoking and alcohol use.

### Survival outcomes

**Figure 3:**
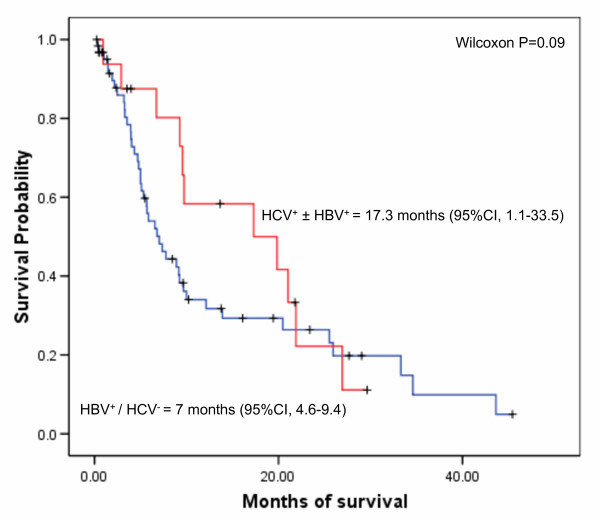
**Kaplan-Meier survival curves for patients with HBV and HCV. HBV^+^/HCV^- ^represents patients with HBsAg^+^/anti-HBc^+^/anti-HCV^- ^or HBsAg^-^/anti-HBc^+^/anti-HCV^-^**. HCV^+ ^± HBV^+ ^represents patients with anti-HCV^+^/HBsAg^-^/anti-HBc^-^, anti-HCV^+^/HBsAg^-^/anti-HBc^+^, or anti-HCV^+^/HBsAg^+^/anti-HBc^+^. Median survival is indicated.

Overall, median survival time for all patients was 9.2 months (95% confidence interval [CI], 6.5–11.9). HCC patients with HCV infection had median survival of 17.3 months (95% CI, 1.1–33.5), while HCC patients with chronic HBV alone had median survival of 7 months (95% CI, 4.6–9.4) (Figure 3), but this difference was not significant (p = 0.09).

**Figure 4:**
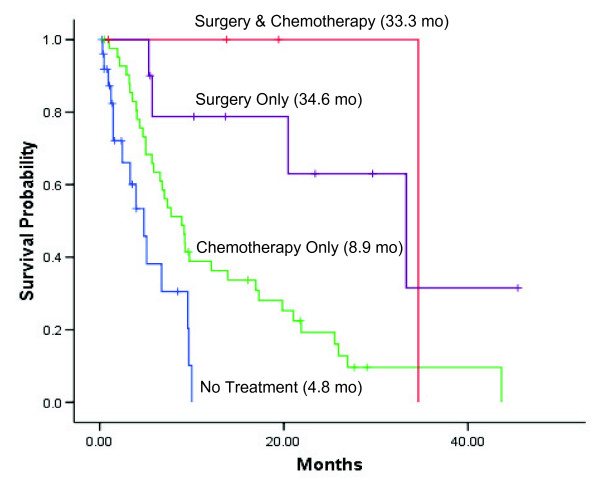
**Kaplan-Meier survival curves stratified by treatment exposure at M. D. Anderson Cancer Center. Median survival is indicated in parentheses**.

Patients with early-stage HCC who underwent surgical resection of the tumor alone at our institution survived the longest (median survival time, 34.6 months; 95% CI, 14.2–52.4); those treated with chemotherapy only at our institution had a median survival time of 8.9 months (95% CI, 6.1–11.7). Adding chemotherapy to surgery did not significantly extend survival relative to that after surgery only. Patients never treated for HCC had the shortest mean survival time: 4.8 months (95% CI, 2.8–6.8) (Figure 4).

### Multivariate analysis of predictors of survival

Results of multivariate Cox regression analyses to identify significant (p ≤ 0.05) predictors of survival are shown in Table 3. We found that males with HCC were 2.4 times as likely to die than females (95% CI = 1.2 – 4.8, p = 0.02). HCC patients with high alpha-fetoprotein levels (≥ 100 ng/mL) were 2.6 times as likely to die than patients with alpha-fetoprotein levels <100 ng/ml (95% CI = 1.4–4.9, p = 0.003). In addition, patients with stage IV disease were 5.6 times as likely to die than patients with stage II or III disease (95%CI = 2–15.3, p = 0.001). Conversely, patients who had received any kind of treatment for HCC prior to or at M. D. Anderson ("ever treated") had an 80% reduction in the risk of death compared with no treatment (95% CI = 0.1–0.4, p = 0.00). We examined the effect of different categories of treatments (chemotherapy, surgery, and other therapies) on survival, but we found no meaningful change to the positive survival effect of "ever treated" in our model (data not shown.)

**Table 3:** Predictors of Survival: Cox Regression Multivariate Analysis

Variable	Hazard Ratio	95% Confidence Interval	p value
Age	0.9	0.9 – 1.02	0.5
Male sex	2.4	1.2 – 4.8	0.02
Cirrhosis	0.9	0.6 – 1.8	0.9
Moderately differentiated tumor	1.9	0.9 – 4.0	0.1
Poorly differentiated tumor	1.1	0.4 – 2.9	0.9
HBsAg^+^	1.4	0.7 – 3.1	0.4
anti-HBc^+^	0.7	0.2 – 2.1	0.5
anti-HCV^+^	1.1	0.4 – 2.5	0.9
Diabetes	1.02	0.5 – 2.3	0.9
Alcohol use	0.6	0.3 – 1.3	0.2
Cigarette smoking	0.6	0.3 – 1.2	0.2
Family history of cancer	0.9	0.5 – 1.6	0.8
Ever treated	0.2	0.1 – 0.4	0.000
AFP ≥ 100	2.6	1.4 – 4.9	0.003
TNM stage III	2.1	0.7 – 6.02	0.2
TNM stage IV	5.6	2.0 – 15.3	0.001

## Discussion

In our series of 82 Asian Americans with HCC treated without transplantation at our tertiary cancer center between 1992 and 2005, we found that the rate of chronic HBV infection was high, at 61%. The rate of chronic HCV infection was significantly lower, at approximately 20%. The prevalence of other risk factors for HCC, such as cigarette use and alcohol abuse, was high, especially among patients with HCV. Survival outcomes were poor overall and in the HBV and HCV subgroups. The major predictors of worse survival were male sex, high alpha-fetoprotein levels, stage IV disease, and lack of treatment for HCC.

Nearly 94% of the patients in our study had previous HBV infection, as evidenced by a positive anti-HBc test result, and 61% of the patients had chronic HBV, as indicated by HBsAg seropositivity. The rate of chronic HBV in our study was lower than the 80% rate reported in a previous study by Hwang et al. of 79 Asian American HCC patients in California [17]. The higher rate of chronic HBV in that study may reflect the overall higher prevalence of chronic HBV among the Asian American population in California, reported to be as high as 8.9% [10]. Our cancer center is a tertiary referral center and sees cancer patients referred from various areas: about 58% are from Texas, 39% are from other states in the US, and 3% are international patients (unpublished institutional data). In another study of 220 Asian and Pacific Islander HCC patients in Hawaii, where the prevalence of chronic HBV may be less than 2% [19], Wong et al. reported that approximately 50% of the study patients had HBV [18], similar to the chronic HBV rate in our study (although the specific HBV test used in the Wong et al. study was not stated).

Our study also showed that the proportion of Asian American HCC patients with previous HBV did not decrease over time. This finding contrasts with the results of a study by Hassan et al., who studied 359 HCC patients seen at our institution during a similar period, 1993–1998, and found that the rate of previous HBV infection decreased over time [20]. Hassan et al. reported HBV infection rates for the overall group and did not break down results by ethnicity [20]. Given the paucity of population-based surveillance data describing HBV infection rates in the United States, our finding of no change in the rate of chronic HBV infection among Asian Americans with HCC has important implications in terms of interventions to reach this underserved group. All Asian Americans must be offered cost-effective HBV screening and vaccination [21]. New and effective anti-HBV medications are available [22,23], and patients found to have chronic HBV must have close surveillance and be considered for treatment. We believe that primary care physicians are the most logical solution to reduce the burden of HBV-associated HCC among Asian Americans because primary care physicians can institute systematic and cost-effective hepatitis screening and treatment for Asian Americans [21].

Although HCV is not the most prevalence risk factor for HCC, our study adds support to the body of evidence indicating that HCV is an important risk factor for HCC among Asian Americans, especially for individuals who do not have chronic HBV. Although our numbers are small, our study revealed the rate of HCV infection to be 20% among Asian American HCC patients, of whom 88% had a negative HBsAg test result. In the previously mentioned study by Hwang et al. in California, 20% of the Asian American HCC patients had a positive HCV result, and most of these patients (69%) had no evidence of HBV [17].

Since the observed prevalence of HBV among patients in our study was high, it is likely that Asian American patients with HCC are relatively less affected than other patients with HCC by other HCC risk factors, such as HCV infection, cigarette smoking, alcohol, and diabetes. Results from our ongoing case-control study where new HCC patients are prospectively enrolled [24,25] indicate that non-Hispanic whites are more exposed to non-virus risk factors than Asian Americans. For example, the prevalence of cigarette smoking, alcohol drinking, and diabetes mellitus among non-Hispanic whites with HCC was 70.4%, 69.4%, and 31.3%, respectively. In our study of Asian American patients with HCC, we found the prevalence of these non-virus risk factors to be lower – 43.9%, 34.1%, and 11%, respectively. Our study findings were similar to those reported previously for Asian HCC patients in Hawaii (32% rate of alcohol use and 48% rate of cigarette use) [18]. Diabetes mellitus, however, was more prevalent in the Hawaii study (29%) than in our study, and this is likely due to the significant prevalence of diabetes in Hawaii, reported to be approximately 6% [26]. It has been shown that the Asian and Pacific Islander population in Hawaii is twice as likely to be diagnosed with diabetes as US Caucasians [27].

The median survival rate in our study of Asian American HCC patients, for whom transplantation was not available and most of whom received palliative therapy, was very poor: 9.2 months. This is lower than the median survival time of 1.56 years reported by Wong et al. in their study of Asians and Pacific Islanders in Hawaii [18]. The difference may be attributed to the higher proportion of stage IV disease in our study (61%) than in Wong et al.'s study (32%). The stage of disease at presentation influences whether surgical options are available. Only 11% of the patients in our study had surgical resection; in contrast, in the study of Wong et al. [18], 54% of patients had stage I or II disease, and 24% of patients underwent liver resection and 7% liver transplantation. This supports the notion that patients with earlier-stage disease treated with surgical treatment options have more favorable survival outcomes [14,15]. Similarly, in a study of 255 HCC patients by Barazani et al. [16] in which 18% of patients with HBV-associated HCC had liver transplantation, the 1-year survival rate was 85%, significantly higher than our 1-year survival rate of approximately 40%. Once HCC is advanced and transplantation is no longer an option, survival is poor.

We believe that our study is the only one to date to examine the impact of treatment, excluding liver transplantation, on the survival of Asian American HCC patients. We found that having received any type of palliative therapy or even liver resection was a predictor of longer survival. This again illustrates that it is critical to implement HBV screening, surveillance, and treatment programs for Asian Americans to ensure that if HCC develops, it will be detected early, when curative therapy options are still available.

Our study also found that elevated alpha-fetoprotein levels and stage IV disease were associated with shorter survival. These findings are similar to findings of Wong et al. [18]. However, whereas we found that chronic HBV was not a significant predictor of survival, Wong et al. found that chronic HBV was associated with worse survival [18]. It is unclear which HBV screening test(s) were used to define HBV disease in the Wong et al. [18] study, and differences in the testing methods may have contributed to the survival differences. Our study does support a previous finding by Hwang et al. [17], who described no significant survival differences between HCC patients with chronic HBV (positive HBsAg) and HCC patients with HCV (positive anti-HCV).

The proportion of patients in our study who had a history of alcohol use, 35%, is similar to the prevalence of 32% reported for Asian HCC patients by Wong et al. [18]. However, in our study, alcohol use was not a significant predictor of survival, whereas in the Wong et al study, it was [18]. The difference may be due to the definition of alcohol use. We defined alcohol use as at least 4 drinks monthly for 6 months during the patient's lifetime, whereas Wong et al. defined alcohol use as 2 drinks daily for 10 years.

We acknowledge that our study had limitations, including its retrospective nature and small sample size. Our study population represents various subcategories of Asian ethnic groups, and their prevalences may be unique because of their country of origin. In addition, the small number of Asian American HCC patients with HCV is problematic, and the related results discussed above should be interpreted with caution. Our institution is a referral center, and it may be difficult to retrospectively ascertain full details of previous treatments that referred patients received. Similarly, we may have underestimated the survival lengths because our analysis began with the treatments at M. D. Anderson; however, we think that this effect is likely minimal since our referral waiting periods are not lengthy. In addition, we think that our study may have underestimated the true effect of HBV, as we did not specifically examine patients with an isolated positive anti-HBc test result. Some such patients may have an undetectable level of HBsAg and may actually be chronically infected. However, we think that this would be a small number [28]. We are aware that comparative studies of the clinical significance of hepatitis and predictors of survival in patients with HCC of different ethnicities would be helpful and could potentially lead to future advances in policy, practice, and research. We are currently conducting such comparative studies of patients treated at our institution, and a longer report is planned for the future. We are also aware that our institution's status as a tertiary referral center may have biased our study population as such centers may attract patients with later-stage disease and more complex cases.

Our findings in this study have important implications for clinical practice. We believe that primary care physicians are the most logical medical providers to break the burden of HBV-associated HCC among Asian Americans. We advocate the use of cost-effective strategies developed by Hutton et al., such as screening all Asian Americans for HBV, treating those who have evidence of chronic HBV, and subsequently screening and vaccinating family members of patients found to have chronic HBV [21]. These strategies will systematically identify patients at risk for HCC and lead them to appropriate secondary cancer prevention. We also advocate close surveillance and treatment of HBV patients according to therapeutic endpoints [23]. We anticipate that significant efforts will be necessary to implement these strategies in daily clinical patient care, and we call for collaboration among national, state, and local community organizations since up to 25% of Asian Americans in some parts of the country do not have a usual place for health care, most likely because of lack of insurance and/or other barriers [29].

In conclusion, we have shown that chronic HBV remains a significant problem among Asian American with HCC referred to our institution and that survival of patients with HCC treated without transplantation at our institution is poor. Early detection of HCC is vital for surgical options to be feasible, and systematic screening of Asian American individuals for HBV will be crucial in improving the outcomes of Asian American patients with HCC. Future cost analyses and quality-of-life studies will be necessary to further elucidate the burden of HCC among special populations and the impact of screening and early detection of this disease.

## Competing interests

The authors declare that they have no competing interests.

## Authors' contributions

JH and MH participated in the conception, design, and coordination of the study. MH performed the statistical analysis. JH drafted the manuscript. All authors critically revised the draft and approved the final manuscript.

## Pre-publication history

The pre-publication history for this paper can be accessed here:

http://www.biomedcentral.com/1471-2407/9/46/prepub
